# Adipose Mesenchymal Stromal Cell-Derived Exosomes Carrying MiR-122-5p Antagonize the Inhibitory Effect of Dihydrotestosterone on Hair Follicles by Targeting the TGF-β1/SMAD3 Signaling Pathway

**DOI:** 10.3390/ijms24065703

**Published:** 2023-03-16

**Authors:** Yunxiao Liang, Xin Tang, Xue Zhang, Cuixiang Cao, Miao Yu, Miaojian Wan

**Affiliations:** 1 Department of Dermato-Venereology, The Third Affiliated Hospital of Sun Yat-sen University, Guangzhou 510000, China; 2 Department of Rehabilitaion Medicine, The Third Affiliated Hospital of Sun Yat-sen University, Guangzhou 510630, China

**Keywords:** androgenic alopecia, adipose-derived stromal cells, exosomes, dermal papilla cells, dihydrotestosterone, TGF-β signaling pathway

## Abstract

Androgenic alopecia (AGA) is the most common type of hair loss, where local high concentrations of dihydrotestosterone (DHT) in the scalp cause progressive shrinkage of the hair follicles, eventually contributing to hair loss. Due to the limitations of existing methods to treat AGA, the use of multi-origin mesenchymal stromal cell-derived exosomes has been proposed. However, the functions and mechanisms of action of exosomes secreted by adipose mesenchymal stromal cells (ADSCs-Exos) in AGA are still unclear. Using Cell Counting Kit-8 (CCK8) analysis, immunofluorescence staining, scratch assays, and Western blotting, it was found that ADSC-Exos contributed to the proliferation, migration, and differentiation of dermal papilla cells (DPCs) and up-regulated the expression of cyclin, β-catenin, versican, and BMP2. ADSC-Exos also mitigated the inhibitory effects of DHT on DPCs and down-regulated transforming growth factor-beta1 (TGF-β1) and its downstream genes. Moreover, high-throughput miRNA sequencing and bioinformatics analysis identified 225 genes that were co-expressed in ADSC-Exos; of these, miR-122-5p was highly enriched and was found by luciferase assays to target SMAD3. ADSC-Exos carrying miR-122-5p antagonized DHT inhibition of hair follicles, up-regulated the expression of β-catenin and versican in vivo and in vitro, restored hair bulb size and dermal thickness, and promoted the normal growth of hair follicles. So, ADSC-Exos enhanced the regeneration of hair follicles in AGA through the action of miR-122-5p and the inhibition of the TGF-β/SMAD3 axis. These results suggest a novel treatment option for the treatment of AGA.

## 1. Introduction

Androgenic alopecia (AGA) is the most common cause disease of chronic hair loss in men and women, with 50% of people over the age of 40 affected by AGA, while affecting 73% of men and 57% of women over the age of 80 [1,2,3]. The patients’ characteristic, self-confidence and quality of life were affected. The pathogenesis of AGA has a genetic tendency and may be determined by multiple genes [4], lifestyle choices (smoking) and the exposed environment (chemical irritants, radiation, pollutants and microorganisms) [5]. The hair follicles (HFs) cycle was shown to be prolonged in the resting phase (telogen) and shortened in the active phase (anagen) in AGA patients. The traditional treatment methods are topical minoxidil, oral finasteride pills and hair transplant; topical and oral treatments do not always produce good results, and sometimes, they cause side effects [6]. Autologous hair transplant is the most effective treatment of AGA in the short term, but it is time-consuming, painful, and limited by the donor area follicles [7].

The development of various of different autologous cell biological techniques, such as autologous stem cells [8,9] and exosomes derived from cells [10,11,12], were reported in the treatment of AGA or hair regeneration. Although different kinds of methods are available to slow or reverse the progression of AGA, its therapy remains challenging [13].

Adipose-derived stromal cells (ADSCs) belong to the mesenchymal stem cells population and were found in the stromal-vascular fraction of fat tissue. Stromal-vascular fraction cells (SVFs) also have been used for many years for autologous applications in plastic surgery [14], wound healing [15], scar repair [16] and treatment of hair loss diseases [17]. The SVFs can be easily isolated from human fat and provide a rich source of ADSCs [18]. ADSCs have been used as a cellular therapeutic approach in AGA [19], knee arthritis [20], skin photoaging [16], wound repair [18], and harmful burn [21]. ADSC-derived proteins may be feasible clinical therapeutic agents for the treatment of hair loss. This is because ADSCs are easy to harvest from fat tissue, which can be taken from body, with the advantages of reduced trauma and fewer ethical issues, together with high yield. So, the cell products of ADSCs may be become alternative therapeutic treatment in hair loss disease. ADSC-derived cytokines, such as platelet-derived growth factor, vascular endothelial growth factor (VEGF) and hepatocyte growth factor (FGF) could activate hair development and promote hair growth in AGA [22].

Exosomes are extracellular vesicles with diameters between 30 and 120 nm that transport microRNAs, proteins, and lipids and function as communication bridges between donor and recipient cells [23,24]. It is well-known that exosomes are harvested from cell-conditioned medium and are derived from cells. The exosomes secreted from adipose-derived stromal cells (ADSC-Exos) have the advantages of being easily isolated and convenient to use, and they do not provoke a significant immune response [25]. Stromal cell exosomes can serve as novel treatment options to repair, regenerate, and rejuvenate skin tissue [26] and have been concerned in nerve repair [27], wound healing [28], and the vascularization of skin flaps [29]. Additionally, it had been reported that ADSC-Exos play an active role in hair loss [30,31]. ADSC-Exos can increase the proliferation and survival of dermal papilla cells (DPCs) and maintain their hair inductivity [31]. Additionally, they are considered the most effective methods in the in vivo and in vitro hair inductivity of DPCs [32]. However, the underlying mechanism of these exosomes on AGA are largely unknown.

MicroRNAs can regulate genes expression by inducing the degradation or translation of targeted mRNA. As an epigenetic regulator, some research focuses on the exosome containing microRNAs from the DPCs and hair growth, which have been reported are related with hair growth [10,33,34]. However, a growing number of researchers have demonstrated positive results between hair growth and ADSC-Exos. However, little is known of the relationship between the exosomes containing microRNA from ADSCs and hair growth, let along with AGA and its mechanism.

DPCs are located at the follicle base and activate follicle development and periodic regeneration during HF growth [35]. DPCs support hair growth and regulate the hair cycle. AGA involves the action of dihydrotestosterone (DHT) on DPCs. DHT decreased the levels of β-catenin and is a positive regulator of hair growth [36,37], and promotes the expression of TGF-β1, which is negative regulator of HFs growth [38]. However, the serum-free conditioned media of ADSCs can protect against the injury which DHT causes to DPCs [39]. In our study, miR-122-5p was highly expressed in ADSC-Exos and delivered to DPCs to regulate its biology. A previous study revealed that miR-122-5p was identified as a potential pro-angiogenic factor that promoted angiogenesis through shifting substrate preference to fatty acids in endothelial cells, activated vascular endothelial growth factor signaling and promoted angiogenesis [40]. In the research of spinal cord injury, the overexpression of miR-122-5p alleviated inflammatory response, reactive oxygen species and apoptosis [41]. Research showed that miR-122-5p has the function of down-regulating the TGF-β/Smad pathway in the regeneration of skeletal muscle repair [42].

To sum up, in our study, we speculated that miR-122-5p in ADSC-Exos may be involved in the process of hair growth through the levels of TGF-β/Smad pathway in DPCs, and may alleviate the inhibitory effect of DHT on HFs. This study aimed to investigate the effect of ADSC-Exos carrying miR-122-5p antagonizes DHT on HFs growth and its underlying mechanism. The results will show that the ADSC-Exos carried with miR-122-5p may represent a new treatment option for the clinical treatment of AGA.

## 2. Results

### 2.1. Isolation and Characterization of ADSCs

**Figure 1:**
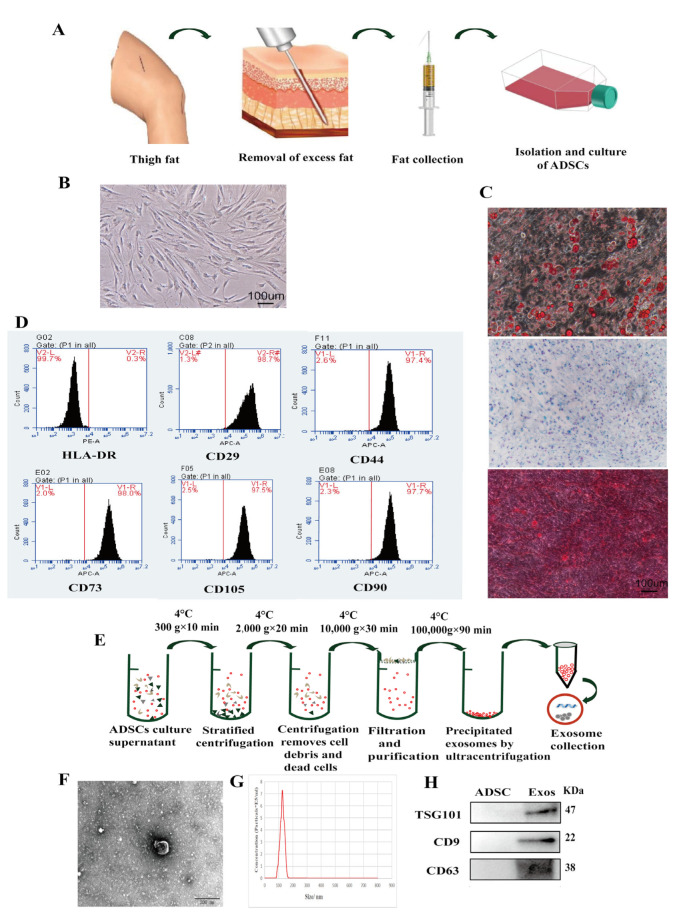
Isolation and characterization of ADSCs. (**A**) Liposuction of human subcutaneous fat and the isolation process of ADSCs. (**B**) Morphology of third-generation ADSCs (Bar = 100um). (**C**) Adipogenic, chondrogenic, and osteogenic differentiation of ADSCs. Adipogenesis was specifically identified by Oil Red O staining, chondrogenesis by Alcian Blue staining, and adipogenesis by Alizarin Red staining. (Bar = 100um). (**D**) Flow cytometric analysis of ADSC-Exo bio-makers. CD29, CD44, CD73, CD90, and CD105 were highly expressed on ADSCs but HLA-DR was rarely expressed. (**E**) The process of ADSC-Exo isolation. (**F**) Morphology of ADSC-Exos observed by transmission electron microscopy (Bar = 200nm). (**G**) The diameter size distribution of ADSC-Exos was detected by nanoparticle tracking analysis (NTA). (**H**) The protein expression of CD9, CD63 and TSG101 was elevated in ADSC-Exos.

The ADSCs isolation steps are shown in Figure 1A. After isolation and three passages in vitro, most of the ADSCs were adherent with a fibroblast-like or spindle-shaped appearance under the microscope (10×) (Figure 1B). ADSCs were induced by adipogenic medium, and it could be seen that the lipid droplets gradually became larger after 21 days with bright red droplets visible under the microscope after Oil Red O staining. Meanwhile, ADSCs were also cultured separately in osteogenic differentiation induction medium or chondrogenic induction medium for approximately three weeks. Mineralization of the cell matrix was visible under the microscope after Alizarin Red S staining, indicating that osteogenesis had occurred, while pellet formation after Alcian Blue staining showed that chondrogenesis had occurred (Figure 1C). To confirm the characterization of the cells, flow cytometry analysis showed strong expression of ADSC biomarkers such as CD29, CD44, CD73, CD90, and CD105 in fourth-generation ADSC cells, compared with DLA-DR (Figure 1D). The isolation process of the exosomes is shown in Figure 1E. Furthermore, the exosomes derived from ADSCs were round- or cup-shaped under EM (Figure 1F) with diameters of 50–150 nm (Figure 1G). Western blot analysis demonstrated that the typical exosomal surface markers such as CD9, CD63 and TSG101 were present (Figure 1H). In addition, we also showed the characterization of ADSC-Exos harvested from conditioned media collected from cells that cultured on exosome-depleted fetal bovine serum (FBS) and full FBS (Figure S1A–C).

### 2.2. ADSC-Exos Promote Hair Growth and Induce HFs to Enter Anagen

To investigate the effect of the ADSC-Exos on HFs, we observed their effects on HFs proliferation in vitro. The results showed that 10 µg/mL of ADSC-Exos could significantly promote HFs proliferation compared with the control group treated with phosphate buffer solution (PBS) (Figure S2A,B). β-catenin in the Wnt signaling pathway has previously been identified as a necessary signaling marker for the growth of HFs [43], and fluorescence immunoassay showed that FITC-labeled fluorescence signals of Ki-67 and β-catenin in HFs treated with 10 µg/mL ADSC-Exos were more obvious (*p* < 0.05) (Figure S2C).

### 2.3. ADSC-Exos Stimulate DPCs Proliferation and Migration

To measure the uptake of ADSC-Exos by DPCs, the exosomes were labeled with the fluorescent dye PKH67 and co-cultured with DPCs in a Petri dish for 4 h, after which the fluorescence signals can be detected in the granule membranes as well as the plasma membranes of DPCs (Figure S3A). The CCK8 assays showed that after incubation with various concentrations of ADSC-Exos, DPCs proliferation reached a relatively optimal level after treatment with 10 µg/mL ADSC-Exos (*p* < 0.01) (Figure S3B,C). The results of the scratch wound assay also revealed that the migration ability of DPCs cultured with ADSC-Exos was enhanced compared with the PBS-treated group (Figure S3D,E). Furthermore, we also performed immunofluorescence staining for Ki-67, which was found to be strongly expressed in DPCs treated with ADSC-Exos, while only a few positive cells were observed in the PBS-treated group (Figure S3F,G). In addition, the expression of versican, β-catenin, and BMP2 were up-regulated in 10ug/mL ADSC-Exos group compared with control group, as were the levels of Cyclin D1 and Cyclin B1 (Figure S3H,I). These results showed that ADSC-Exos promoted the proliferation and migration of DPCs.

### 2.4. ADSC-Exos Counteracted the Inhibitory Effect of DHT on DPCs

**Figure 2:**
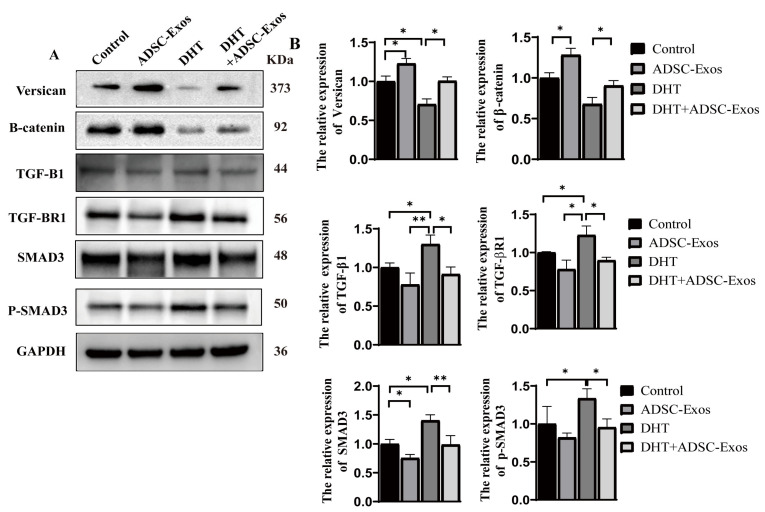
ADSC-Exo mitigated the inhibitory effect of DHT on DPCs. (**A**) The protein expression of the TGF-β/SMAD axis, its downstream genes, and proliferation-regulating genes in DPCs induced by DHT (10^−5^ mol/L). (**B**) Relative expression levels of proteins. Normal human DPCs treated with PBS were used as control. Data are represented as means ± SEM. * *p* < 0.05, ** *p* < 0.01.

The effectiveness of ADSC-Exos on the proliferation of HFs/DPCs has been clari- fied. To explore whether ADSC-Exos can counteract the inhibitory effect of DHT (10^−5^ mol/L) on DPCs, we analyzed the effects of ADSC-Exos on the TGF-β/SMAD signaling induced by DHT, together with proliferation biomarker levels by Western blotting (Figure 2A,B). The results showed that the expression of versican and β-catenin were down-regulated in DHT treated group compared with control group, which indicatig that DHT could inhibit the proliferation of DPCs. However, ADSC-Exos showed the opposite trend, which exerts its pro-proliferation effect on DPCs. When ADSC-Exos and DHT act together on DPCs, compared with DHT group, the expression of SMAD3 and p-SMAD3 in the ADSC-Exos+DHT group showed a downward trend, while β-catenin and verican showed an increasing trend. The level of β-catenin, verican and SMAD3 and p-SMAD3 in ADSC-Exos+DHT group were no significant difference compared with the control group. Similarly, we also found that DHT down-regulated BCL2 and up-regulated Bax expression, while ADSC-Exos played the opposite regulatory role and reversed DHT-induced BCL2 and Bax expression levels. (Figure S4). These results indicated that ADSC-Exos could mitigate the effects of DHT on TGF-β/SMAD protein levels. Our analysis suggested that ADSC-Exos potentially antagonize DHT-induced cell apoptosis or follicle development disorder. However, the specific mechanisms by which the exosomes down-regulate TGF-β signaling remain unclear.

### 2.5. Screening and Functional Analysis of Target Genes

**Figure 3:**
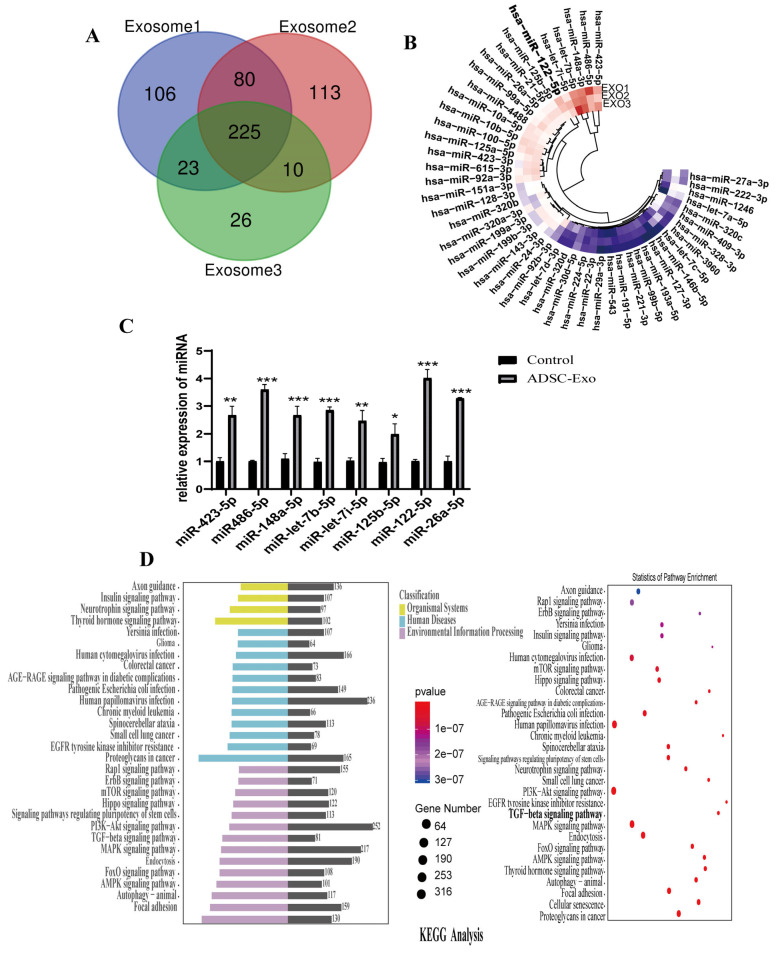
Identification and screening of differentially expressed miRNAs in ADSC-Exos. (**A**) Three exosome samples were performed from three patients with the same baseline and extracted by gradient ultracentrifugation. A total of 225 miRNAs were identified by miRNA sequencing. (**B**) Heatmap and clustering analysis of the top 50 miRNAs contained in the ADSC-Exos. MiRNA expression levels are indicated by colors: red represents high values, and blue represents low. (**C**) qRT-PCR was performed to verify the expression of the top eight miRNAs in DPCs and DPCs-treated with ADSC-Exos. The data represent the means ± SD. DPCs treated with PBS were used as control group. * *p* < 0.05, ** *p* < 0.01, *** *p*< 0.001. (**D**) KEGG analysis shows the predicted target pathways of genes enriched in ADSC-Exos and the statistics of pathway enrichment.

In this study, we sought to explore the miRNA expression profiles of ADSC-Exos and performed functional and pathway enrichment analyses. A total of 225 miRNAs were detected in the sample consensus expression (Figure 3A, Additional Table S1), and the relative expression levels are shown in the heatmap (Figure 3B). We then selected the eight most enriched miRNAs for further verification using semi-quantitative RT-PCR. The results showed that the expression of miR-122-5p was the highest of all the selected microRNAs (Figure 3C). Meanwhile, KEGG pathway analysis showed that miR-122-5p regulates the TGF-β signaling pathway (Figure 3D). This suggested that the exosomes could down-regulate TGF-β signaling induced by DHT, with miR-122-5p likely targeting the pathway to promote hair regeneration. Nevertheless, the details of the mechanism require further clarification.

### 2.6. miR-122-5p Targets and Negatively Regulates SMAD3

**Figure 4:**
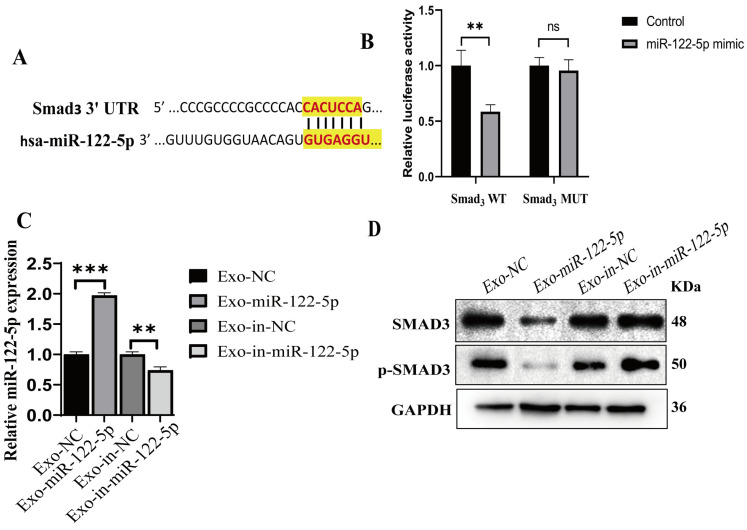
miR-122-5p targets and negatively regulates SMAD3. (**A**) Binding site prediction of miR-122-5p in SMAD3 3′-UTR. (**B**) Luciferase analysis showing miR-122-5p targeting SMAD3. (**C**) The expression of miR-122-5p in Exo-miR-122-5p and Exo-in-miR-122-5p. (**D**) Protein expression of SMAD3 and p-SMAD3 in DPCs treated with Exo-miR-122-5p and Exo-in-miR-122-5p. ** *p* < 0.01, *** *p* < 0.001.

To investigate the function of miR-122-5p, we hypothesized that the binding of the miRNA to its putative target (SMAD3) can inhibit the TGF-β signaling pathway (Figure 4A). Given the proliferative effect of miR-122-5p, we investigated its potential target genes, especially those associated with the TGF-β/SMAD3 signaling pathway. Bioinformatics analysis using the online tools TargetScan and MiRwalk indicated that SMAD3 might be the target of miR-122-5p. In luciferase assays, plasmids containing the wild-type 3 ‘UTR of SMAD3 and the predicted binding site of miR-122-5p, or the mutant 3′ UTR without the binding site were transfected into DPCs. The result showed that the fluorescence decreased when miR-122-5p was bound to the 3′ UTR binding site of SMAD3. However, the fluorescence of the latter did not change significantly (Figure 4B). The quantitative analysis of exosomes collected after transfection demonstrated that miR-122-5p was highly expressed in Exo-miR-122-5p (Figure 4C). Moreover, the expression levels of SMAD3 and p-SMAD3 in Exo-miR-122-5p group were lower than Exo-NC group. This showed that the Exo-miR-122-5p mimic significantly reduced the expression of SMAD3 and p-SAMD3 after transfection (Figure 4D).

### 2.7. ADSC-Exo-miR-122-5p Counteracted the Inhibitory Effect of DHT on HFs in C57BL/6 Mice

**Figure 5:**
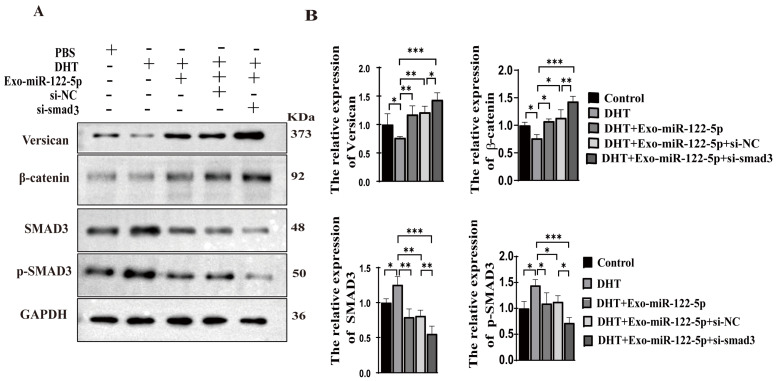
Exo-122-5p targeting SMAD3 mitigates the inhibition of DHT on DPCs. (**A**) Immunoblot analysis and quantification of protein expression of versican, β-catenin, SMAD3, and p-SMAD3 in DHT-induced DPCs treated with Si-SMAD3 and Exo-miR-122-5p. (**B**) Quantification of protein expression (versican, β-catenin, SMAD3, and p-SAMD3). The data represent the means ± SD. Normal DPCs treated with PBS were used as control, *n* = 3, * *p* < 0.05, ** *p* < 0.01, *** *p*< 0.001.

We further investigated whether TGF-β/SAMD3 plays a role in the protection against the effects of DHT. The results showed that the expression of SMAD3 and p-SMAD3 were down-regulated in the group treated with Exo-miR-122-5p, while the levels of β-catenin and versican were increased compared with the control group (Figure 5A,B). After DHT pretreatment, DPCs were incubated with Exo-miR-122-5p, and it was found that this intervention down-regulated the expression of SMAD3 and p-SMAD3 and enhanced the expression of β-catenin and versican (Figure 5A,B), thus indicating that Exo-miR-122-5p could inhibit the TGF-β pathway in DPCs and counteract the inhibitory effects of DHT on DPCs. In addition, we further explored the role of SMAD3 in the TGF-β signaling pathway. We transfected SMAD3 siRNA into cells to simulate the role of Exo-miR-122-5p. The results showed that both SMAD3 and p-SMAD3 levels decreased, while the expression of β-catenin and versican increased compared with the control group (Figure 5A,B). However, when co-cultured with Exo-miR-122-5p, the proliferation and induction of DPCs induced by DHT were restored, as the expression levels of β-catenin and versican were similar to those in the control group. While knockdown of SMAD3 resulted in reduced expression of SMAD3 and p-SMAD3, it is worth noting that both β-catenin and versican levels were significantly increased in this group. These results suggested that Exo-miR-122-5p targeted SMAD3 to down-regulate TGF-β signaling and to restore the proliferation and inducibility of DPCs.

### 2.8. Inhibition of TGF-β/SMAD Signaling Enhances the Protective Effects of ADSC-Exo-miR-122-5p in DHT-Induced HFs

**Figure 6:**
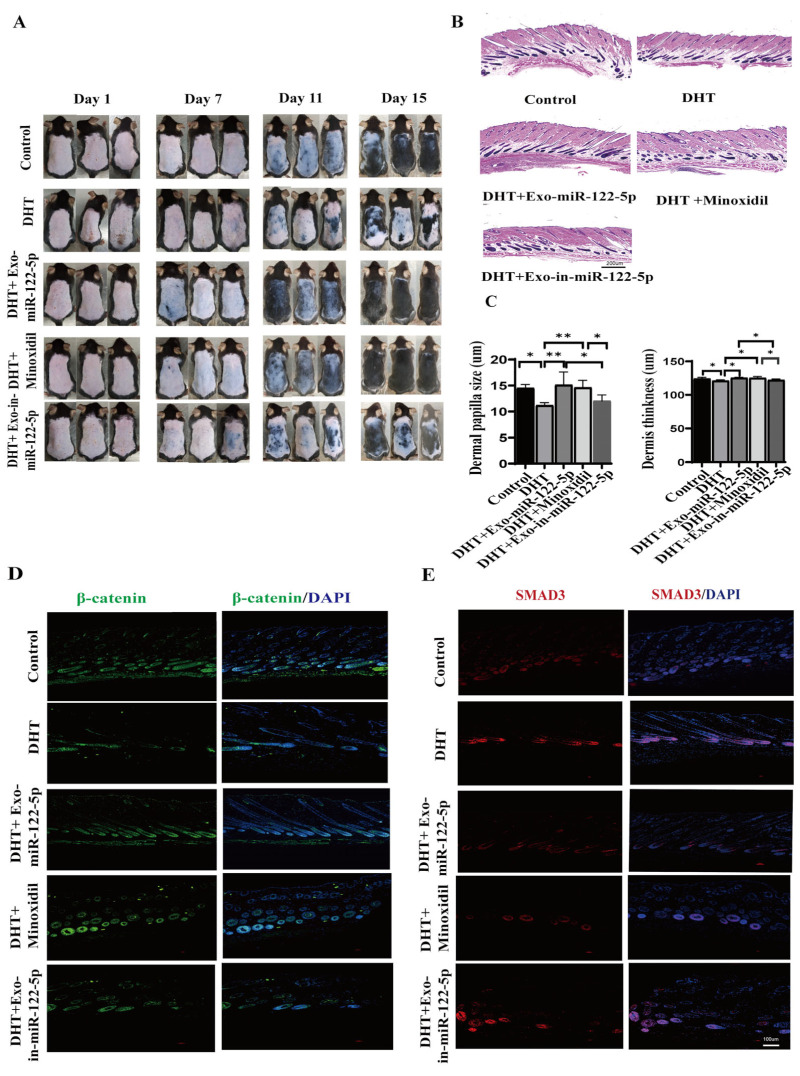
Exo-miR-122-5p mitigates the inhibitory effect of DHT on HFs in C57BL/6 mice. (**A**) All C57BL/6 mice were subcutaneously injected with 200 µL of solution containing DHT (10−5 mol/L) or/and Exosomes (50 µg/mL) after shaving, and the dorsal hair growth was observed and photographed every two days. The dorsal skin of C57BL/6 mice began to turn grayish-brown on day 7. The dorsal hair coverage of minoxidil and Exos-miR-122-5p treated mice were significantly more than those in the DHT group on day 15, and the DHT + Exo-in-miR-122-5p group showed lower hair coverage compared with the control group. (**B**) Hematoxylin and eosin (H&E) staining of C57BL/6 mouse dorsal skin treated with NS, DHT, DHT+Exo-miR-122-5p and DHT+Exo-in-miR-122-5p. (Bar = 200 um.) (**C**) Hair bulbs and dermal thickness of C57BL/6 mouse dorsal skin in each group shown by H&E staining. (**D**) Immunofluorescence images of β-catenin and (**E**) the expression of SMAD3 on C57BL/6 mouse dorsal skin from different treatment groups. (Bar = 100 µm) The data represent the means ± SD. * *p* < 0.05, ** *p* < 0.01.

To investigate whether ADSC-Exo-miR-122-5p reverses the inhibitory effect of DHT on HFs, we depilated the dorsal hair of mice with depilatory cream to cause simultaneous entry of the HFs into catagen. The skin of the back in the catagen phase was pink (Figure 6A). After subcutaneous injection of 200 µL Exo-miR-122-5p and DHT, respectively, we found that the HFs of the mice in the two groups began to grow after seven days. By day 11, the dorsal skin of the Exo-miR-122-5p + DHT and minoxidil + DHT groups gradually become gray, indicating that the HFs had entered the anagen phase, while the dorsal skin of the mice in the DHT group was still pink. On day 15, compared with the DHT group, Exo-miR-122-5p + DHT and minoxidil + DHT groups showed larger fields range of hair coverage and were exuberant with the control group. Compared with the DHT groups, the Exo-miR-122-5p + DHT group showed a significant difference in HFs proliferation. The skin tissue from the backs of the mice were collected for histological analysis. This showed that the dermal thickness of the Exo-miR-122-5p + DHT group was greater than that of DHT group (Figure 6B,C), indicating that Exo-miR-122-5p could provide suitable culture conditions for HFs proliferation, and the size of the hair bulbs in this group was larger than those in the DHT group (*p* < 0.01) (Figure 6B,D); furthermore, the DHT + Exo-in-miR-122-5p group presented lower hair coverage, smaller dermal papilla and thinner thickness compared with the control group (Figure 6A–C). In addition, the fluorescence immunoassay results showed that compared with the DHT group, the expression of β-catenin in DHT+ Exo-miR-122-5p was upregulated, and was similar to control group, illustrating that Exo-miR-122-5p enhanced the growth of HFs even after the treatment with high DHT concentrations (Figure 6D), and it also down-regulated the expression of SMAD3 (Figure 6E). Otherwise, the expression of β-catenin and SMAD3 in the DHT + Exo-in-miR-122-5p group were similar to DHT group, illustrating that the Exo-in-miR-122-5p could not antagonize the inhibitory effect of DHT on HFs (Figure 6D,E). It has been reported that β-catenin is the main initiator of HF growth and plays a critical role in regulating the hair cycle and promoting hair growth. DHT significantly blocks hair development; thus, the expression of SMAD3 would be up-regulated, and the expression of β-catenin would be significantly down-regulated, indicating that antagonism existed between the TGF-β and Wnt pathways. The above evidence showed that Exo-miR-122-5p was delivered to HF cells, alleviated the inhibitory effect of DHT, and down-regulated TGF-β signaling by targeting SMAD3 to restore HFs proliferation.

## 3. Discussion

Given the limited efficacy of traditional treatments of AGA, we investigated and successfully prepared a new biological product, ADSC-Exos. In the present study, we evaluated the effects of ADSC-Exos on the proliferation and cycle change in DPCs/HFs in vitro and explore the therapeutic effect of ADSC-Exos on HFs induced by DHT. The findings showed that ADSC-Exos could promote the proliferation of HFs/DPCs and alleviated the DHT-induced inhibition of DPCs and down-regulated the TGF-B/SMAD signaling pathway. MiR-122-5p is highly enriched in ADSC-Exos, which was found to specifically reduce SMAD3 expression in DPCs after the treatment of DHT. It was further confirmed that this effect was mainly exerted by exosomes transferring to the DPCs. These results suggest that miR-122-5p derived from ADSC-Exos may represent a promising strategy for the treatment of AGA.

Due to the distribution of dermal papillae in the dermis and the subcutaneous fat layer, the periodic changes and regeneration of HFs are inseparable from the surrounding molecular environment [10]. A preliminary study observed that ADSC-Exos could promote HFs regeneration in mice [30], cell migration, proliferation, and the inhibited cell apoptosis of fibroblast and keratinocytes cells [44,45], although there is no clear conclusion regarding AGA. In our study, we found that the growth of HFs and proliferation of DPCs could be promoted after being incubated with ADSC-Exos, and the expression of ki-67 and cell migration increased compared with the control group. ADSC-Exos was also found to increase the expression of versican, β-catenin, BMP2 and cyclin. It would thus be expected, in principle, that increasing the ADSC-Exos concentration would result in the stronger promotion of DPCs proliferation. However, it was found that the response plateaued at 10 µg/mL, which differs from Enshell’s report [10] and suggests that additional factors may influence ADSC-Exos activity. The discrepancy may due to the differences in the viability of the stromal cells or the isolation methods [46].

It was also found that the ADSC-Exos could counteract the inhibitory effects of DHT on HFs. DHT is known to sensitize ARs in DPCs and to activate the TGF-β signaling pathway, and significant upregulation of phosphorylation SMAD2 and SMAD3 has also been observed [47], which is consistent with our results. Pretreatment of DPCs with DHT followed by treatment with ADSC-Exos resulted in significant down-regulation of TGF-B1, TGF-BR1, SMAD3 and p-SMAD3, in comparison with cells treated with DHT alone, while the expression of β-catenin and versican was restored to a relatively normal level compared with the control group. Meanwhile, the expression of anti-apoptotic protein BCL2 decreased and the apoptotic protein Bax increased in the DPCs induced by DHT. Under the action of ADSC-Exos, the apoptosis of DPCs could be restored. Studies have shown that TGF-B1 and TGF-B2 have long been identified as the major mediators in the development of AGA [48,49], and the down expression of BMP2 was observed in the DPCs of AGA patients, which would compromise HFs integrity and hair shaft differentiation [50]. Recent evidence has shown that the exosomes derived from human umbilical cord mesenchymal could promote wound healing by inhibiting TGF-β signaling [51]. The existing evidence indicated that MSC-Exos induce the proliferation of DPCs and secretion of VEGF, which is conducive to the development of HFs [52], it also accelerates the transition from the resting phase to the growth phase and stimulates the expression of Shh and β-catenin [53]. In addition, it is not difficult to get inspiration from the application of MSC-Exos in the treatment of wound, the TGF-β signaling pathway and the PI3K/Akt pathway could be regulated by MSC-Exos to accelerate the wound healing and improved skin regeneration [54,55,56]. These findings are in accordance with our experiment results and other treatment involving AGA [57].

MiRNAs within exosomes form an important component of paracrine signaling to mediate intercellular communication and functional interaction [53]. Further investigation of the mechanism by which ADSC-Exos mitigate the effects of DHT showed that miR-122-5p was a highly enriched component of the ADSC-Exos miRNA expression profile and that it modulated the TGF-β signaling pathway by targeting SMAD3 to reduce the effects of DHT on HFs growth, thus restoring their normal growth. One study has shown that miR-122-5p is an effective angiogenic factor that could activate VEGF and promote angiogenesis [40]. It is worth mentioning that the core mechanism of minoxidil in hair loss is to promote the expression of VEGF and promote hair regeneration [58]. In addition, it has been found that miR-122-5p suppresses the differentiation and collagen synthesis of TGF-β1-stimulated cardiac fibroblasts, reducing the expression of SMAD3 and p-SMAD3 [59], which is consistent with our results. Additionally, the overexpression of miR-122-5p in keratinocytes was reported to promote its proliferation [60]. The TGF-β signaling pathway is down-regulated in anagen, which would enhance the expression of Ki-67 and β-catenin [61]. SMAD3, as a downstream messenger and functional gene in TGF-β1 signaling, usually binds to SMAD2 and SMAD4 to enter the nucleus when the TGF-β signaling pathway is activated, which is consistent with our study. Our results also revealed that the expression of SMAD3 and p-SMAD3 was decreased in the Exo-miR-122-5p-treated cells compared with the cells treated with DHT only; correspondingly, the expression of versican and B-catenin in DPCs restored in the DHT+ Exo-miR-122-5p group. The directional knockdown of SMAD3 further illustrates the importance of TGF-β in AGA and the effectiveness of miR-122-5p.

C57BL/6 mice are ideal for studying AGA. Our previous research showed that DHT with appropriate concentration can inhibit the growth of HFs [62]. We thus simulated the pathogenesis of AGA with DHT (10^−5^ mol/L). Compared with the control group, DHT significantly activated TGF-β signaling and enhanced the expression of SMAD3. The mice skin showed smaller dermal papilla size, thinner dermis and lower dorsal hair coverage. After treating with Exo-miR-122-5p, compared with the control group, the hair coverage rate on the back of mice in this group almost returned to the normal level, even seem to be more exuberant than control group, and the expression of SMAD3 in the dorsal skin of DHT-induced mice was significantly reduced compared with the DHT group. To our knowledge, extracellular vesicles contain various types of miRNAs, lipids, and proteins. In our experiments, we mainly analyzed miR-122-5p in ADSC-Exos, which exhibits a prominent role in resisting androgenetic alopecia. Meanwhile, the expression of β-catenin in the hair matrix cells and inner root sheath cells was also increased, indicating that DPCs may have a specific regulatory effect on the proliferation of these cells, especially in the endosomal environment of HFs. DPCs regulate the proliferation and migration of outer root sheath cells or hair matrix cells through the paracrine pathway [61,63]. ADSC-Exos could promote proliferation and migration in keratinocytes and endothelial cells [64]. Similarly, the β-catenin and Versican levels in the DHT + Exo-miR-122-5p group gradually returned to normal compared with the control group when TGF-β signaling was blocked. The injection of exosomes may be more advantageous than commercial minoxidil, because subcutaneous injection of ADSC-Exos can prevent the dormant state of DPCs. However, compared with the control group, the hair coverage was less, dermal papilla size was smaller and the thickness was thinner in DHT + Exo-in-miR-122-5p group, which could not reverse the inhibition effect of DHT on HFs. Thus, we concluded that ADSC-Exos carrying miR-122-5p could mitigate DHT inhibition of HFs by targeting SMAD3. However, a limitation to this study is that we focused only on miR-122-5p, which was highly expressed in ADSC-Exos, and the contribution of other miRNAs can’t be excluded.

## 4. Methods

### 4.1. Isolation and Culture of Human HFs

The ethics committee of the Third Affiliated Hospital of University approved this research [(2022)-02-098-01]. Occipital scalp tissue was obtained from healthy adult men undergoing cosmetic surgery and was washed with normal saline to clear blood clots. The excess adipose tissue under the dermis was removed with sterile ophthalmic scissors. Single HF was isolated with the help of an anatomical microscope (Nikon, Tokyo, Japan). HFs in anagen were selected and cultured in 24-well plates. The medium was serum-free Williams’ medium (Gibco, Waltham, MA, USA), and 2 mM HEPES (Gibco), 2 mM/L-glutamine (Gibco), 10 mg/L insulin (Gibco), 10 µg/L hydrocortisone (Apexbio, Houston, TX, USA), and antibiotics (100 mg/L streptomycin and 100,000 U/mL penicillin), (Gibco) and were cultured, at 37 °C, in an atmosphere of 5% CO_2_. After 24 h of culture, HFs that had grown to 0.3–0.5 mm were randomly divided into four groups. The lengths of the follicles were photographed and measured every two days.

### 4.2. DPCs Culture

DPCs (Jian Daoshou, Nanjing, China) were cultured in Dulbecco Modified Eagle Medium (DMEM) (Gibco) supplemented with 1% penicillin-streptomycin, 10% fetal-bovine-serum (Gibco), and 1 ng/mL fibroblast growth factor (Jian Daoshou). DPCs were maintained in a 37 °C atmosphere with 5% CO_2_. DPCs between passages 3 and 5 were used in this experiment.

### 4.3. Isolation and Identification of ADSCs

Adipose tissue was obtained from the thighs of patients undergoing plastic revision surgery. After the removal of visible blood vessels and excessive anadesma, the adipose tissue was cut into small pieces of no more than 0.5 mm and was digested with 0.2% type I collagenase (Sigma-Aldrich, St Louis, MO, USA) in a centrifuge tube, at 37 °C, for 20–30 min, after which it was thoroughly shaken to separate the stromal cells from the turbid liquid. Complete medium containing 10% FBS was used to halt digestion. After centrifugation at 1000× *g* for 5 min, at room temperature, the upper adipose tissue was removed, and the tissue fragments and supernatant were removed with a 70 µm filter. The cell precipitate was obtained after further centrifugation at 1000× *g* for 3 min, and the cells were cultured in DMEM supplemented with 10% FBS and 1% penicillin/streptomycin, at 37 °C, in a 5% CO_2_ atmosphere.

For adipogenic and osteogenic differentiation, the cells were induced with adipogenic, osteogenic, and chondrogenesis medium, resulting in the formation of adipocytes after 21 d and the formation of osteocytes and chondrocytes after 23 d. The differentiated cells were then washed with PBS, fixed in 4% paraformaldehyde (Macklin, Shanghai, China) for 30 min, stained with Oil Red O (Oricell, Guangzhou, China) for 20 min, stained with hematoxylin (Cyagen, Beijing, China) for 2 min, and observed under the microscope (Olympus Optical, Tokyo, Japan). Similarly, osteogenesis and chondrogenesis were induced in strict accordance with the operating instructions provided by Oricell. After culture in adipogenic and chondrogenic induction medium for approximately three weeks, the ADSCs were stained with Alizarin Red (Oricell) and Alcian Blue (Oricell) and observed under the microscope.

For flow cytometry, ADSCs were harvested at the third passage by digestion with trypsin. The cells were then resuspended in 100 µL PBS and incubated with the relevant antibodies, namely, anti-HLA-DR, anti-CD29, anti-CD44, anti-CD73, anti-CD105, and anti-CD90 (Abcam, Cambridge, UK; 10^6^ cells/1 µL) on ice for 5–10 min. The labeled cells were then analyzed on a flow cytometer (Cytek, Fremont, CA, USA), and the data were analyzed and processed with FlowJo 10 software (FlowJo vro 10.6.1, LLC, Ashland, OR, USA).

### 4.4. Extraction and Identification of ADSC-Deprived Exosomes

ADSCs reaching 70% confluence were cultured in DMEM supplemented with 10% exosomes depleted FBS, and after 24 h culture, ADSC-Exos were extracted from culture supernatants by gradient ultracentrifugation; at the same time, we also collected ADSC-Exos of complete culture medium for comparison. The ADSCs culture supernatant was collected and centrifuged at 4 °C and 300× *g* 10 min, 2000× *g* 20 min, and 10,000× *g* 30 min to remove cell fragments and debris, and the large extracellular vesicles were filtered and removed through a 0.22 µm filter. The remaining supernatant was centrifuged at 100,000× *g* for 90 min to enrich the exosomes. Finally, after removing the supernatant, the exosomes were resuspended in PBS and stored, at −80 °C. It was worth noting that the centrifuge tubes need to be replaced with new tubes after each centrifugation.

For electron microscopic analysis, the exosomes collected by gradient centrifugation were fixed in 1% glutaraldehyde for 5 min, dehydrated with the same volume of 1% nitrous oxide, stained with 1% phosphotungstic acid for 5 min, and were observed under transmission electron microscopy (Hitachi, Tokyo, Japan).

For NTA, 10 µL resuspended exosomes were diluted 1000 times with PBS, and the concentration and particle size distribution of exosomes were measured using nanoparticle size tracking analyzer (ViewSizer 3000, Irvine, CA, USA).

### 4.5. Cellular Uptake and Tracing of ADSC-Exos

ADSC-Exos in PBS were labeled with the green-fluorescent dye PKH67 (Sigma) according to the provided instructions [65]. The labeled exosomes were collected by ultracentrifugation at 10 0000× *g* 90 min and were incubated with DPCs, at 37 °C, for 4 h. After fixing the cells with 4% paraformaldehyde for about 10 min, the nuclei were counterstained with 4′,6-diamidino-2-phenylindole (DAPI). All procedures were conducted in the dark. The cells were observed under a fluorescence microscope (Nikon, Tokyo, Japan).

### 4.6. HF Treatment

After 24 h of hair follicle culture, HFs in the growth stage were randomly divided into four groups and were co-cultured with different concentrations of ADSC-Exos for seven days. The hair lengths were measured and photographed every two days. On day 7, the growth of the HFs was evaluated by Ki-67 staining and hair stem length.

### 4.7. Cell Proliferation Assay/Cell Counting Kit-8

DPCs proliferation was measured using the CCK-8 (Dojindo, Shanghai, China). The total of 5 × 10^3^ DPCs in a volume of 100 µL per well were inoculated into a 96-well plate, treated with different concentrations of ADSC-Exos for 24 h, 48 h, or 72 h and cultured, at 37 °C, with 5% CO_2_. After that, 10 μL CCK-8 reagent was placed in each well and incubated, at 37 °C, for 2 h. The absorbance at 450 nm was measured by a microplate reader (BioTek elx-808, Winooski, VT, USA). The mean values of all wells were statistically analyzed, and the experiment was repeated three times.

### 4.8. Cell Migration Scratch Test In Vitro

DPCs were inoculated at 2 × 10^5^ cells per well and cultured in 6-well plates. The cells were cultured until nearly confluent in complete medium, after which scratches were made on the cells with a sterile 200 µL pipette tip, and suspended necrotic cells were washed off with PBS. The cells were incubated with different concentrations of ADSC-Exos for 24 h and 48 h and photographed under an inverted microscope. The scratch area was quantified using Image J software. Cell migration was calculated as the mobility rate (%) = (initial area—residual area)/initial area × 100%.

### 4.9. Cell Transfection and Exosome Editing

Passage-3 ADSCs were cultured in DMEM with 10% FBS. When the cells were approximately 70% confluent, they were infected with lentivirus loaded with the lentivector constructs of the pre-miRNA-122-5p and anti-miRNA-122-5p clusters (Genechem, Shanghai, China; referred to as Exo-miR-122-5p and Exo-in-miR-122-5p, respectively) or the corresponding empty lentivector (Genechem, Shanghai, China; referred to Exo-NC and Exo-in-NC, respectively), as previously described [66]. Subsequently, 2 µg/mL puromycin was added for 3–4 days to produce a stable transduction cell line. The exosomes were harvested from the culture media of these ADSCs, respectively. Specifically, when the cells reached 80% confluence, the normal culture medium was replaced with an exosome-free medium. The culture supernatant was retained, and the exosomes were collected by centrifugation as described above. The luciferase experiment can be performed according to the previous research protocol [66]. The expression of miR-122-5p in the exosomes was assessed by a semi-quantitative PCR assay.

For siRNA knockdown, 60%-confluent DPCs were transfected with 50 nM si-SMAD3 using Lipofectamine 3000 (Thermo Fisher Scientific, Waltham, MA, USA). After 24 to 48 h of culture in double antibody-free medium, the cells were harvested for further analysis.

### 4.10. Hematoxylin and Eosin Staining and Immunofluorescence Staining

For H&E staining, skin tissue sections from C57BL/6 mice were fixed with 4% paraformaldehyde, cut into 4 µm sections, and stained with H&E (BBL-009, BASMEDTSCI, Beijing, China). For immunofluorescence staining, cell climbing tablets and dewaxed hydrated sections were permeabilized with 0.5% Triton X-100 (diluted with PBS), at room temperature, for 10 min and blocked with 1% BSA for 15 min, after which they were incubated with the appropriate primary antibodies and incubated, at 4 °C, overnight. Then, corresponding fluorescent labeled secondary antibodies (Affinity Biosciences, Cincinnati, OH, USA) was used to probe the primary antibody binding. The primary antibodies included anti-Ki-67 (ab92742,1:300, Abcam, Cambridge, UK), anti-β-catenin (ab223075, 1:300, Abcam), and anti-SMAD3 (AF6362, 1:300, Affinity Biosciences). After washing with PBS, the nuclei were counterstained with DAPI for about 5–8 min. The images of cells and sections were observed and photographed under the fluorescence microscope.

### 4.11. RNA Isolation and Quantitative Real Time-PCR

Total RNA was isolated from cells and tissues using a total RNA Extraction Kit (Haigene, Harbin, China) according to the manufacturer’s instructions. Five hundred nanograms of RNA was used for reverse-transcription to cDNA with a TaqMan microRNA Reverse Transcription Kit (Takara, Dalian, China). To analyze miR-122-5p expression, qRT-PCR was performed using the FQTM miRNA poly (A) QRT PCR SYBR Kit (Enzy Valley, Guangzhou, China). The primers are shown in Table S2 (Supplementary Materials). The PCR reaction conditions were pre-denaturation, at 95 °C, for 10 min, followed by 40 reaction cycles (95 °C, 10 s), annealing (60 °C, 20 s), and extension (70 °C, 10 s). The relative expression of target genes was calculated using the 2^−ΔΔCt^ method. All reactions were carried out in triplicate. The amount of miR-122-5p was determined by standardizing to U6.

### 4.12. miRNA Sequencing of ADSC-Exos and Bioinformatics Analysis

For miRNA sequencing, total RNA was extracted from the ADSC-Exos using an Extraction Kit (Haigene, Harbin, China). A Nanodrop 2000 bioanalyzer (Thermo Fisher Scientific) was used for RNA quantification. cDNA libraries were generated by reverse-transcription using an Illumina TruSeq RNA Sample Preparation Kit (RS-122-2001; Illumina, San Diego, CA, USA) and the distribution of the template sizes was checked. The libraries were sequenced on an Illumina HiSeq 2500 system to produce paired-end readings. The raw read files were obtained with the CASAVA tool v1.8 (Illumina). The quality of the sequence reads was checked by the webservers FastQC. To obtain miRNA sequences, other ncRNA sequences, such as small nuclear RNA (snRNA), small nuclear RNA (snRNA), and small nuclear RNA (snRNA) were filtered out. The remaining sequences were analyzed with miRBase 22.0 (http://www.mirbase.org (accessed on 5 September 2020)) and miRWalk (http://mirwalk.uni-hd.de (accessed on 7 September 2020)) to detect the miRNAs. A heatmap of the top 50 most enriched miRNAs in the ADSC-Exos was generated, and their target genes were analyzed and predicted using the MiRanda database (http://www.microrna.org/microrna (accessed on 5 September 2020)). The Kyoto Encyclopedia of Genes and Genomes (KEGG, http://www.kegg.jp, (accessed on 9 September 2020)) pathway enrichment analysis was used to identify signaling pathways that could be regulated by the sequenced miRNAs.

### 4.13. Western Blotting

Total protein was isolated from the cells using a whole protein extraction kit (KeyGEN, KGP2100, Nanjing, China), using lysis buffer containing 1 mM PMSF, 5 U/mL phosphatase inhibitor, and 1 µL/mL protease inhibitor. Twenty micrograms of protein was loaded onto 10% SDS-PAGE gels and then transferred to polyvinylidene fluoride membranes (Pierce, Waltham, MA, USA). The membranes were blocked with 5% skimmed milk, at room temperature, followed by incubation with the primary antibodies overnight at 4 °C. The antibodies included the Abcam antibodies anti-TSG101 (1:1000, ab83), anti-CD9 (1:1000, ab236630), anti-CD63 (1:1000, ab134045), anti-versican (1:1000,ab177480), anti-β-catenin (1:1000, ab223075), anti-cyclin D1 (1:1000, ab134175), anti-cyclin B1 (1:1000, ab32053), anti-BMP2 (1:1000, ab214821), and anti-GAPDH (1:1000, ab181602), as well as antibodies purchased from Affinity Biosciences, namely, anti-SAMD3 (AF6362, 1:1000, Affinity Biosciences), anti-p-SMAD3 (1:1000, ab52903), anti-BCL2 (1:1000, AF6139) and anti-Bax (1:1000, AF0083). The membranes were then incubated with horseradish peroxidase-conjugated goat anti-rabbit IgG (1:10 000, ab150077), at room temperature, for 1h. The protein bands were visualized using Kf003 Extremely Sensitive ECL chemiluminescence buffer (Affinity Biosciences) and the ChemiDoc Touch Imaging System (Bio-Rad, Hercules, CA, USA). The band densities were measured using BioRad Quality One and Image J software.

### 4.14. Hair Growth In Vivo Study

Six-week-old male C57BL/6 mice were purchased from the Animal Core Facility of Guangzhou Animal Research Institute (Guangzhou, China), and maintained under controlled temperature (23 °C ± 1 °C) and humidity (55 ± 10%) conditions. After a one-week static observation period, all mice were randomly divided into control group (*n* = 6), DHT group (*n* = 6), DHT + Exo-miR-122-5p group (*n* = 6), DHT + Minoxidil group (*n* = 6) and DHT + Exo-in-miR-122-5p group (*n* = 6). A solution of PBS (200 µL), DHT (10^−5^ mol/L, 200 µL), and Exo-miR-122-5p (50 ug/mL, 200 µL) was injected subcutaneously at six points on the related dorsal skin once a day starting from the first day after depilation. All mice were observed and photographed every two days. After 15 days, skin samples were collected for further experimental analysis.

### 4.15. Statistical Analysis

Data were expressed as means ±SD. The differences between groups were calculated by Student’s *t*-test, and the comparisons among the different interventions were analyzed by one-way analysis of variance, followed by Dunnett’s post hoc test. *p* < 0.05 was considered statistically significant. The above analysis was carried out using SPSS18.0 software (IBM R18.0.0, Armonk, New York, NY, USA).

## 5. Conclusions

Our study provided further insights into the regulatory functions of ADSC-Exos cargos and their application in AGA. ADSC-Exos promoted HF growth, DPCs proliferation, and mitigated the inhibitory effect of DHT on DPCs by inhibiting the TGF-β signaling pathway. In addition, miR-122-5p, which was highly enriched in ADSC-Exos, up-regulated the expression of β-catenin and versican by targeting SMAD3 in vivo and in vitro, restored hair bulb size and dermal thickness, and accelerated the normal growth of HFs. The findings of this study provide a potential novel approach for further treatment strategies of AGA.

## Data Availability

The raw data supporting the conclusion of this article will be made available by the authors, without undue reservation.

## Supplementary Materials

The supporting information can be downloaded at https://www.mdpi.com/article/10.3390/ijms24065703/s1.

## Abbreviations

ADSCs-Exos	Adipose mesenchymal stromal cell-derived exosomes
AGA	Androgenic alopecia
DAPI	4′,6-Diamidino-2-phenylindole
DHT	Dihydrotestosterone
DMEM	Dulbecco’s modified Eagle’s medium
DPCs	Dermal papilla cells
FBS	Fetal bovine serum
H&E	Hematoxylin and eosin
HFs	Hair follicles
MSCs	Mesenchymal stromal cells
NTA	Nanoparticle tracking analysis
SVF	Stromal-vascular fraction
SVFs	Stromal-vascular fraction cells
TGF-β1	Transforming growth factor β1
VEGF	Vascular endothelial growth factor
