# Supplementary material for: Adipose Mesenchymal Stromal Cell-Derived Exosomes Carrying MiR-122-5p Antagonize the Inhibitory Effect of Dihydrotestosterone on Hair Follicles by Targeting the TGF-β1/SMAD3 Signaling Pathway
Source: Int J Mol Sci. 2023 Mar 16;24(6):5703. doi: 10.3390/ijms24065703 (PMC10059832; doi:10.3390/ijms24065703)
Supplement: Supplementary file 1 [file ijms-24-05703-s001.zip › ijms-2142049-supplementary.pdf]

Supplementary Materials for

Adipose Mesenchymal Stem Cells Derived Exosomes Carrying MiR-122-5p Antagonize the Inhibitory Effect of Dihydrotestosterone on Hair Follicles by Targeting the TGF-β1/SMAD3 Signaling Pathway

Yunxiao Liang[1†], Xin Tang[1†], Xue Zhang[2], Cuixiang Cao[1], MiaoYu[1], Miaojian Wan[1*]

* Correspondence: E-mail: wanmj@mail.sysu.edu.cn

This file includes:

Figures: Figure S1- Figure S4
Tables: Additional table S1 and Additional table S2

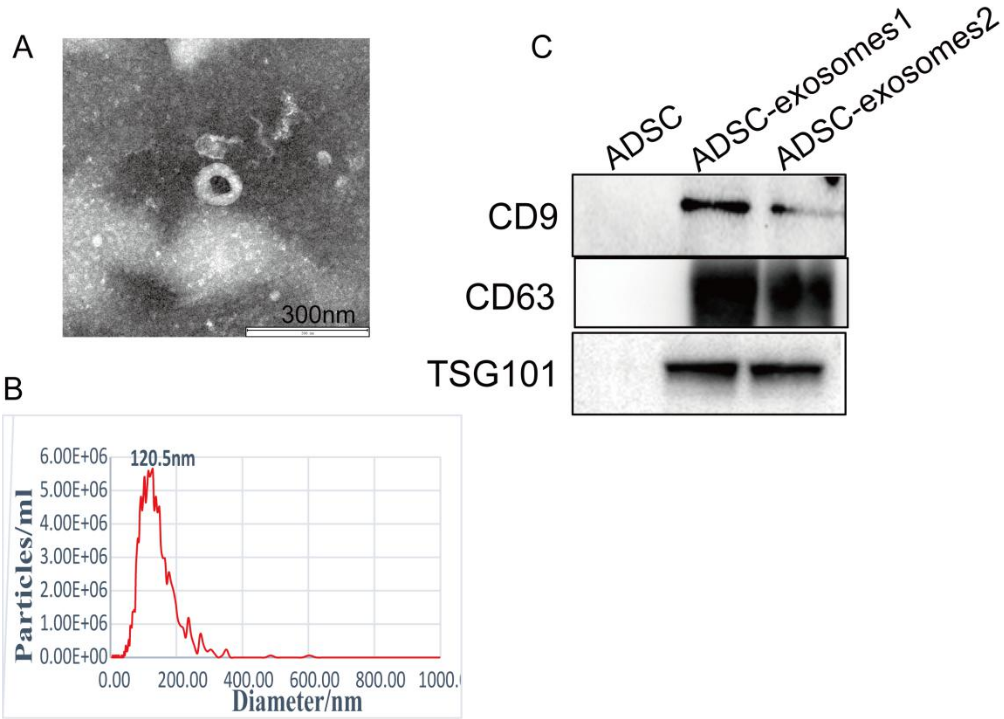

Figure. S1

The ADSC-Exosomes were havested from conditioned media collected from cells that cultured on both exosome-depleted FBS (ADSC-exosomes1) and FBS (ADSC-exosomes2).(A) The Morphology of ADSC-Exosomes were deprived from conditioned media added with full FBS and observed by transmission electron microscopy (Bar=300nm). (B) Particle size concentration and distribution of ADSC-Exosomes from adding full FBS conditioned media detected by nanoparticle tracking analysis. (C) Western blotting was conducted to assess the protein levels of CD9,CD63 and TSG101 in terms with ADSC-Exosomes havested from conditioned media collected frocells that cultured on both exosome-depleted FBS and FBS.

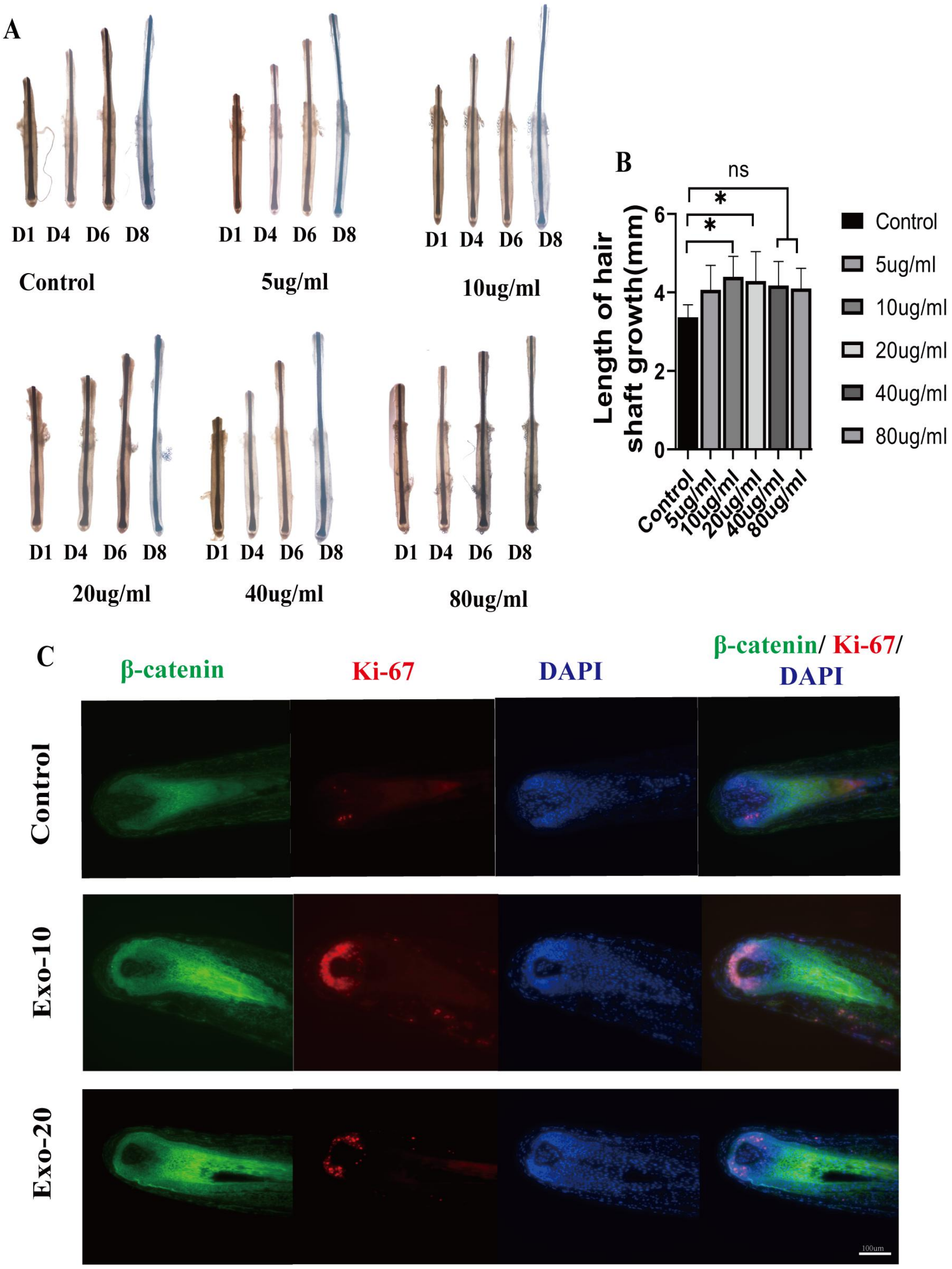

Figure S2

Growth-promoting effect of Exos on hair follicles cultured in vitro.

Continuous photomicrographs of hair follicles co-cultured with different concentrations of Exos. Hair follicles in the growth period were randomly divided into groups and photographed every two days. (B) Statistical analysis was conducted on the measured length of hair growth before entering catagen. (C) Immunofluorescence staining of Ki67 in the hair matrix and ORS regions and β-catenin in the hair matrix, IRS, and ORS regions of HFs on day 8 (Bar=100 μm). *$p<0.05$.

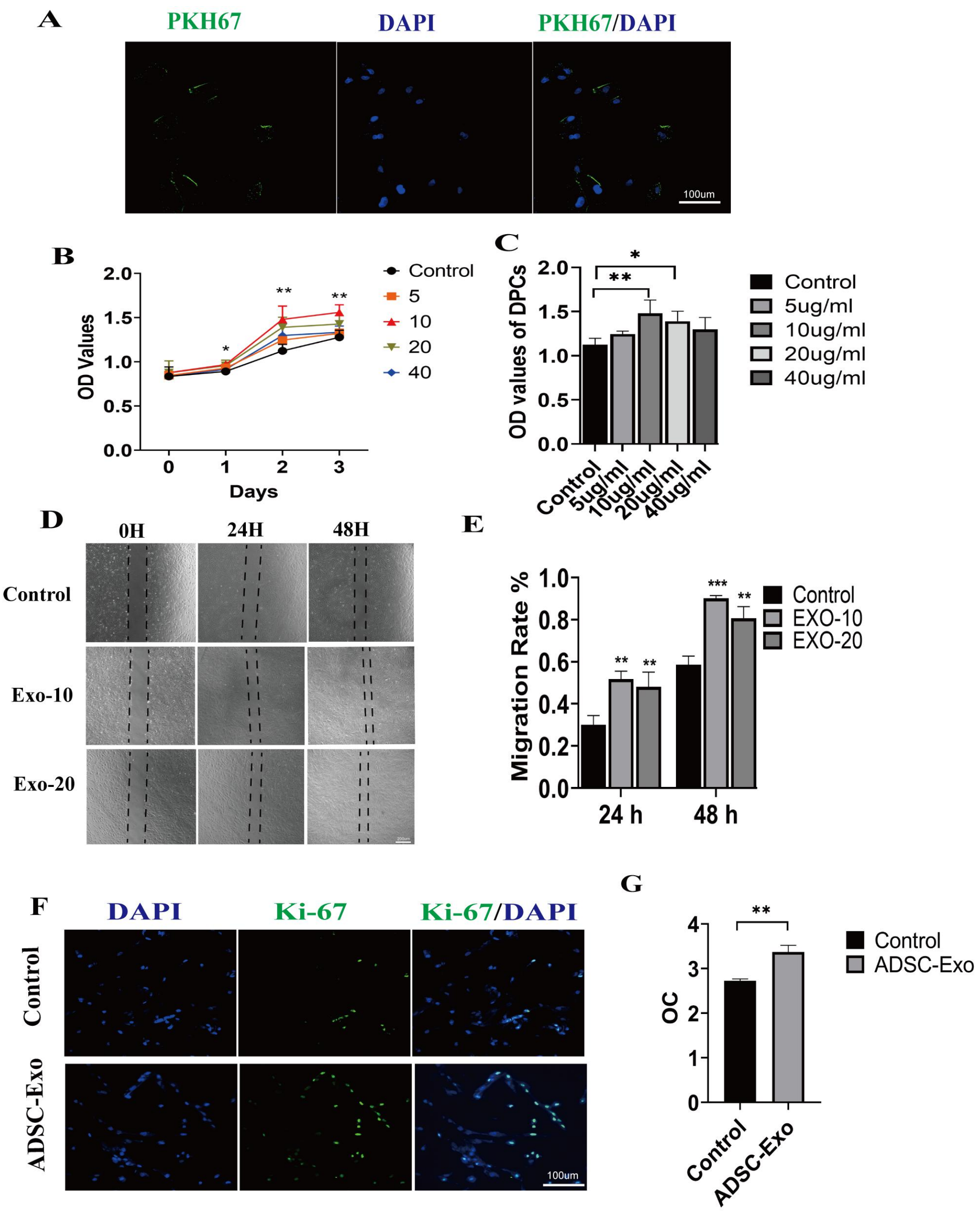

A
PKH67
DAPI
PKH67/DAPI
100um
B
OD Values
Days
Control
5
10
20
40
C
OD values of DPCs
Control
5ug/ml
10ug/ml
20ug/ml
40ug/ml
D
0H
24H
48H
Control
Exo-10
Exo-20
E
Migration Rate %
24 h
48 h
Control
EXO-10
EXO-20
F
DAPI
Ki-67
Ki-67/DAPI
Control
ADSC-Exo
100um
G
OC
Control
ADSC-Exo

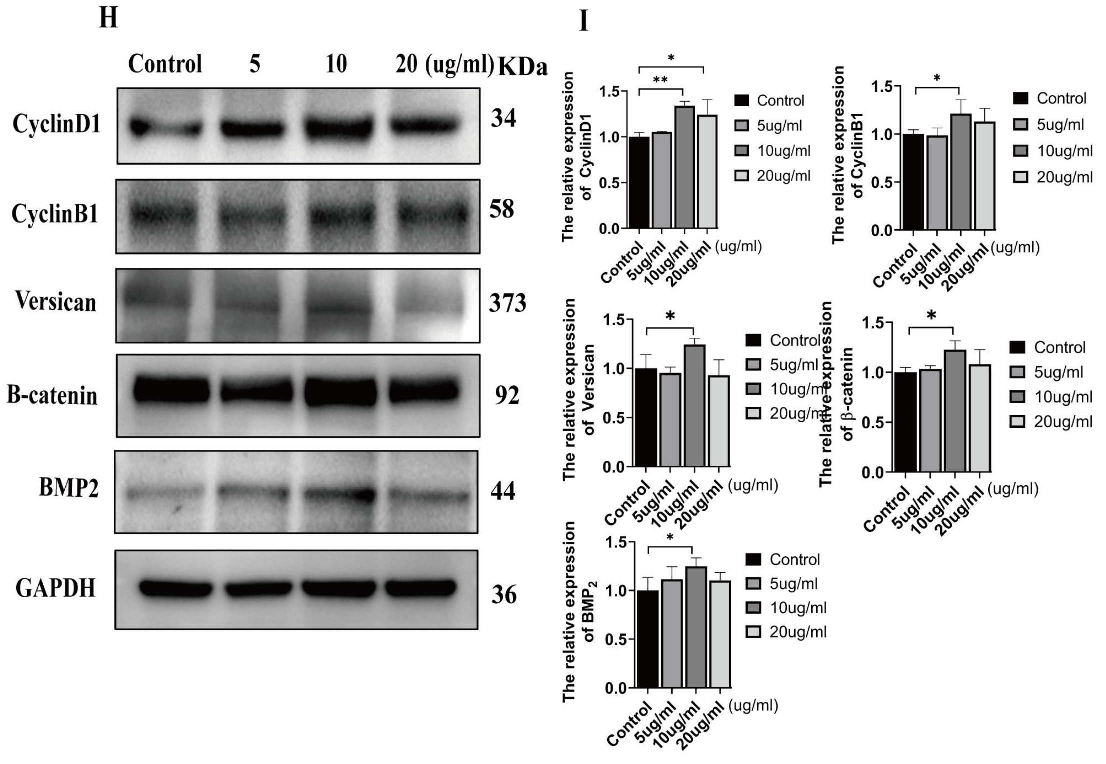

Figure. S3

ADSC-Exos stimulate DPCs proliferation and migration.

(A)PKH67-labeled ADSC-Exos internalized by DPCs (Bar=100 μm). (B, C) DPCs were treated with ADSC-Exos, and cell proliferation was detected by CCK-8 analysis. After incubation for 1 day, the number of DPCs increased and was highly significant after 2 days (Bar=200 μm). (D, E) DPCs were incubated with an appropriate concentration of ADSC-Exos and the mobility rate was detected by the cell migration scratch test (Bar=100 μm). (F, G） Immunofluorescence staining of Ki67 in DPCs treated with ADSC-Exos and the quantitative statistical analysis of the percentage of Ki67+. (Bar=100um) (H, I) The relative protein expression of cyclin and growth regulatory proteins in DPCs treated with ADSC-Exos were analyzed by Western blotting. DPCs treated with PBS were used as controls. Data are represented as means ±SD. *p SD. *p<0.05, **p <0.01.

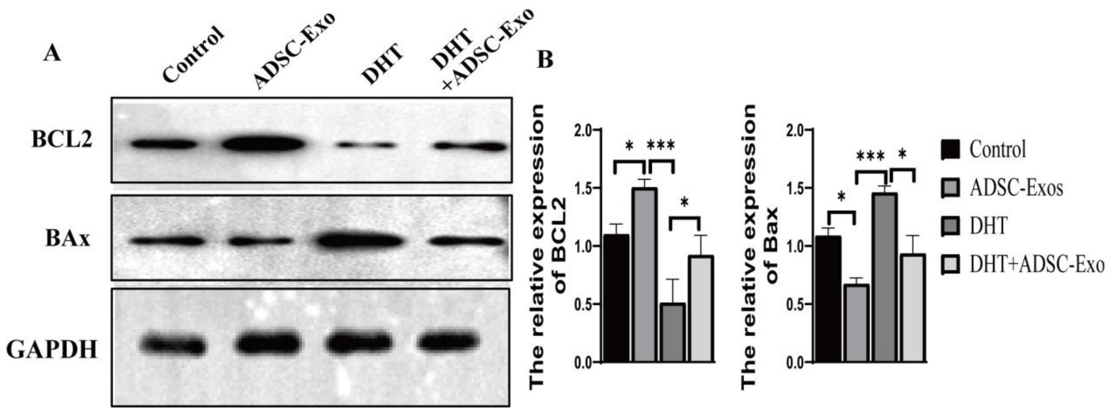

Figure. S4

ADSC-Exo mitigated the inhibitory effect of DHT(10^{-5}mol/L) on DPCs.

In the DPCs induced by DHT（10^{-5} mol/L）, ADSC-Exos can upregulate BCL2 expression and downregulate Bax expression.Data are represented as means ± SEM. *$p < 0.05$, **$p < 0.01$, ***$P < 0.001$

Additional Table S1
The miRNAs were detected in the sample consensus expression.

tracking_id	EX01	EX02	EX03
hsa-miR-148a-3p	80140.38	73007.03	91707.28
hsa-let-7b-5p	74258.07	65710.51	110957.99
hsa-miR-486-5p	47157.39	103028.62	47608.22
hsa-let-7i-5p	47077.07	22878.23	33102.7
hsa-miR-122-5p	39946.61	45241.24	19705.34
hsa-miR-423-5p	35868.54	52746.64	64813.1
hsa-miR-99a-5p	32037.62	16055.14	19463.82
hsa-miR-100-5p	31197.29	23449.14	27078.86
hsa-miR-615-3p	28651.58	15372.83	33599.95
hsa-miR-21-5p	27854.5	16347.56	17560.06
hsa-miR-125b-5p	27218.08	16013.36	17531.64
hsa-miR-10b-5p	26952.38	31595.07	23157.69
hsa-miR-423-3p	25308.79	10471.35	30062.37
hsa-miR-10a-5p	24165.69	24451.72	16707.63
hsa-miR-26a-5p	23652.84	13868.96	16395.08
hsa-miR-125a-5p	21230.71	14620.9	27149.9
hsa-miR-4488	19902.25	25147.95	14718.63
hsa-miR-199b-3p	19889.88	9399.15	13851.99
hsa-miR-199a-3p	19889.88	9399.15	13851.99
hsa-miR-92a-3p	18524.36	20970.55	27547.71
hsa-miR-143-3p	18345.17	12866.39	13823.58
hsa-miR-24-3p	16856.06	9385.23	11110
hsa-miR-1246	14112.62	11195.43	3935.39
hsa-miR-27a-3p	12234.23	6837.01	6435.85
hsa-miR-128-3p	12172.44	9510.55	12829.07
hsa-miR-151a-3p	12036.5	9162.43	12374.44
hsa-let-7a-5p	10621.53	9970.06	2003.22
hsa-miR-222-3p	10034.54	6280.03	6421.64
hsa-miR-3960	9867.71	3898.91	5384.52
hsa-miR-320a-3p	9101.53	17712.18	12630.17
hsa-miR-320b	8601.03	9608.02	17702.13
hsa-let-7c-5p	7668.02	2478.59	3367.1
hsa-miR-92b-3p	7124.28	7435.77	23768.59
hsa-miR-409-3p	6988.34	6516.74	10911.11
hsa-miR-320c	6110.94	5305.3	14235.58
hsa-miR-30d-5p	5919.39	6850.94	6052.25
hsa-miR-328-3p	5425.08	3592.56	10470.68
hsa-miR-22-3p	4671.25	4372.35	4006.42

hsa-miR-543	4306.7	2506.44	2429.43
hsa-let-7d-3p	4214.01	3425.47	6677.37
hsa-miR-193a-5p	4084.26	2840.63	4915.68
hsa-miR-127-3p	4016.29	1991.23	5540.8
hsa-miR-29a-3p	3960.68	1949.45	2713.57
hsa-miR-7704	3960.68	710.16	866.64
hsa-miR-320d	3830.92	3481.17	8240.16
hsa-miR-99b-5p	3806.2	2701.39	4617.33
hsa-miR-221-3p	3719.7	2715.31	4390.02
hsa-miR-199a-5p	3657.92	835.48	3267.64
hsa-miR-224-5p	3552.87	4205.25	4461.05
hsa-miR-191-5p	3219.21	2743.16	2543.08
hsa-miR-10396a-5p	3033.84	696.24	255.72
hsa-miR-424-3p	2879.37	1406.39	2628.33
hsa-miR-25-3p	2681.64	2771.01	2315.77
hsa-miR-370-3p	2632.21	236.72	2159.49
hsa-miR-27b-3p	2514.81	1225.37	923.47
hsa-miR-218-5p	2471.56	1587.42	312.56
hsa-miR-146b-5p	2465.38	2158.32	4034.84
hsa-let-7g-5p	2422.13	1197.52	1136.57
hsa-miR-4516	2168.8	543.06	2514.67
hsa-let-7f-5p	2107.01	2214.02	795.6
hsa-miR-181a-5p	2063.76	1643.12	994.5
hsa-miR-125b-1-3p	1946.35	1643.11	2401.01
hsa-miR-1290	1915.46	3244.45	738.77
hsa-miR-30c-5p	1828.96	1420.32	1648.04
hsa-miR-6529-5p	1637.41	1601.34	1719.07
hsa-miR-381-3p	1575.62	1169.67	3054.54
hsa-miR-619-5p	1557.08	570.91	539.87
hsa-miR-10396b-5p	1526.19	348.12	127.86
hsa-miR-1291	1421.15	167.1	767.19
hsa-miR-4508	1408.79	654.46	255.73
hsa-miR-27a-5p	1384.07	654.46	1292.85
hsa-let-7b-3p	1371.72	821.56	2429.43
hsa-miR-3195	1371.72	153.17	752.98
hsa-miR-485-5p	1353.18	1294.99	2273.15
hsa-miR-214-3p	1328.46	863.33	1846.93
hsa-miR-532-5p	1279.03	1002.58	539.87
hsa-miR-196a-5p	1235.78	682.31	539.87
hsa-miR-382-5p	1186.35	431.66	653.53
hsa-miR-4466	1180.17	654.46	4787.82
hsa-miR-155-5p	1143.1	584.84	198.9
hsa-miR-146a-5p	1056.59	459.51	170.49

hsa-miR-30a-5p	1031.88	1086.12	1093.95
hsa-miR-941	1019.5	1392.45	4191.1
hsa-miR-193b-5p	994.8	1531.71	2969.3
hsa-miR-503-5p	982.45	473.44	1960.59
hsa-miR-493-3p	976.27	487.36	483.04
hsa-miR-92b-5p	963.91	459.51	1875.35
hsa-miR-1307-3p	951.55	626.61	2315.77
hsa-miR-197-3p	920.66	696.23	1378.1
hsa-miR-432-5p	914.48	584.84	724.57
hsa-miR-101-3p	877.4	919.02	397.8
hsa-miR-126-3p	834.15	1127.9	994.5
hsa-miR-152-3p	834.15	375.97	582.49
hsa-miR-181b-5p	815.62	417.74	340.98
hsa-miR-654-3p	735.29	556.99	1150.78
hsa-miR-4497	735.29	111.4	269.94
hsa-let-7a-3p	729.12	668.38	852.44
hsa-miR-744-5p	704.4	612.69	824.02
hsa-miR-186-5p	642.61	501.29	525.67
hsa-miR-28-3p	611.71	431.66	696.15
hsa-miR-192-5p	605.53	487.36	596.7
hsa-miR-484	593.17	403.82	568.29
hsa-miR-22-5p	587	445.59	142.07
hsa-miR-148b-3p	562.28	696.23	653.53
hsa-miR-1228-5p	543.74	55.7	1676.45
hsa-miR-574-3p	531.39	362.04	1065.54
hsa-miR-493-5p	519.03	306.34	951.88
hsa-miR-296-3p	494.31	167.1	1903.76
hsa-miR-214-5p	488.13	445.59	355.18
hsa-miR-671-3p	488.13	334.19	937.67
hsa-miR-625-3p	469.6	334.19	966.09
hsa-miR-24-2-5p	469.6	97.47	241.52
hsa-miR-103a-3p	451.06	779.78	142.08
hsa-miR-30a-3p	432.52	222.79	426.22
hsa-miR-134-5p	420.17	320.27	397.8
hsa-miR-485-3p	413.99	724.08	1008.71
hsa-miR-654-5p	407.81	236.72	312.56
hsa-miR-26b-5p	401.63	111.4	412.01
hsa-miR-221-5p	395.45	153.17	511.46
hsa-miR-378a-3p	370.73	738.01	497.25
hsa-miR-23a-3p	370.73	222.79	142.07
hsa-miR-495-3p	364.56	236.72	468.84
hsa-miR-130b-5p	364.56	153.17	284.14
hsa-miR-299-3p	364.56	139.25	596.7

hsa-miR-99b-3p	327.48	125.32	525.67
hsa-miR-10400-5p	308.95	459.51	142.07
hsa-miR-145-3p	308.95	431.66	227.31
hsa-miR-7706	296.59	389.89	156.28
hsa-let-7e-5p	290.41	181.02	156.28
hsa-miR-125a-3p	290.41	139.25	71.04
hsa-miR-9901	278.05	292.42	56.83
hsa-miR-106b-3p	259.51	334.19	142.07
hsa-miR-7-5p	259.5	1002.57	653.53
hsa-miR-140-3p	240.98	877.25	198.9
hsa-miR-1180-3p	240.98	389.89	198.9
hsa-miR-342-5p	240.98	27.85	56.83
hsa-miR-379-5p	222.44	97.47	113.66
hsa-miR-574-5p	222.44	55.7	426.22
hsa-miR-365b-3p	222.44	27.84	880.84
hsa-miR-365a-3p	222.44	27.84	880.84
hsa-miR-4492	210.08	41.77	156.28
hsa-miR-210-3p	203.9	125.32	14.21
hsa-miR-323a-3p	191.55	97.47	426.22
hsa-miR-199b-5p	185.37	13.92	312.56
hsa-miR-129-5p	185.36	556.98	369.38
hsa-miR-93-5p	179.19	487.36	71.04
hsa-miR-17-5p	173.01	125.32	14.21
hsa-let-7d-5p	166.83	264.57	99.45
hsa-miR-132-3p	160.65	97.47	184.69
hsa-miR-342-3p	154.47	208.87	213.11
hsa-miR-10a-3p	154.47	69.62	28.41
hsa-miR-20a-5p	148.29	292.42	28.41
hsa-miR-615-5p	148.29	55.7	397.8
hsa-miR-30e-5p	142.11	334.19	14.21
hsa-miR-365a-5p	135.94	167.1	468.84
hsa-miR-455-5p	135.94	125.32	1491.75
hsa-miR-149-5p	135.94	125.32	596.7
hsa-miR-29b-3p	135.94	27.84	56.82
hsa-miR-142-5p	129.76	724.08	142.07
hsa-miR-1468-5p	129.76	153.17	113.66
hsa-miR-16-2-3p	117.4	1281.07	85.24
hsa-miR-629-5p	117.4	473.44	184.69
hsa-miR-20b-5p	117.4	306.34	14.21
hsa-miR-361-3p	117.4	222.79	355.18
hsa-miR-412-5p	117.4	181.02	14.21
hsa-miR-664a-5p	117.4	55.7	28.41
hsa-miR-194-5p	105.04	445.58	28.42

hsa-miR-433-3p	105.04	250.64	156.28
hsa-miR-760	105.04	153.17	28.41
hsa-miR-411-5p	105.04	111.4	113.66
hsa-miR-4324	105.04	69.62	99.45
hsa-miR-452-5p	105.04	13.92	184.69
hsa-miR-98-5p	92.68	83.55	170.49
hsa-miR-125b-2-3p	92.68	41.77	14.21
hsa-miR-330-3p	92.68	13.92	85.24
hsa-miR-340-5p	86.5	111.4	142.07
hsa-miR-6089	86.5	27.84	85.24
hsa-miR-10401-3p	86.5	13.92	355.18
hsa-miR-1908-5p	86.5	13.92	127.86
hsa-miR-2110	80.33	139.25	71.04
hsa-miR-365b-5p	80.33	55.7	99.45
hsa-miR-3615	74.15	946.88	170.49
hsa-miR-4671-5p	74.15	55.7	170.49
hsa-miR-665	74.15	55.7	142.07
hsa-miR-204-3p	74.15	55.7	127.86
hsa-miR-25-5p	74.15	27.85	56.83
hsa-miR-4800-3p	67.97	69.62	99.45
hsa-miR-1249-3p	67.97	55.7	42.62
hsa-miR-10b-3p	67.97	27.85	198.9
hsa-miR-330-5p	61.79	13.92	71.04
hsa-miR-23a-5p	61.79	13.92	71.04
hsa-miR-329-3p	61.78	194.94	340.98
hsa-miR-5100	55.61	13.92	42.62
hsa-miR-339-3p	49.43	139.25	56.83
hsa-miR-320e	49.43	97.47	14.21
hsa-miR-339-5p	49.43	69.62	14.21
hsa-miR-1301-3p	49.43	13.92	269.94
hsa-miR-1271-5p	49.43	13.92	14.21
hsa-miR-1260b	43.25	55.7	28.41
hsa-miR-501-3p	37.07	153.17	14.21
hsa-miR-584-5p	37.07	41.77	14.21
hsa-miR-675-5p	37.07	13.92	99.45
hsa-miR-323b-3p	30.89	83.55	56.83
hsa-miR-34b-3p	30.89	41.77	156.28
hsa-miR-28-5p	30.89	27.85	99.45
hsa-miR-1260a	30.89	27.85	28.41
hsa-miR-6858-5p	30.89	13.92	170.49
hsa-miR-140-5p	24.72	83.55	113.66
hsa-miR-378i	24.72	41.77	28.41
hsa-miR-6750-5p	24.72	13.92	14.21

hsa-miR-4429	24.72	13.92	14.21
hsa-miR-204-5p	18.54	55.7	284.14
hsa-miR-6131	18.54	41.77	28.41
hsa-miR-34a-5p	18.54	41.77	14.21
hsa-miR-106b-5p	18.54	27.85	170.49
hsa-miR-3158-3p	12.36	306.34	227.32
hsa-miR-182-5p	12.36	55.7	42.62
hsa-miR-200c-3p	12.36	41.77	56.83
hsa-miR-326	12.36	13.92	28.41
hsa-miR-200b-3p	12.36	13.92	14.21
hsa-miR-183-5p	6.18	208.87	14.21
hsa-miR-337-3p	6.18	55.7	71.04
hsa-miR-1268a	6.18	13.92	113.66
hsa-miR-193b-3p	6.18	13.92	56.83
hsa-miR-331-3p	6.18	13.92	56.83

Additional Table S2

The eight most enriched miRNAs in ADSC-Exos and their primer sequences used for qRT-PCR

miRNA	Primer Sequence (5 ‘-3’)
hsa-miR-423-5p	AGGGGCAGAGAGCGAGACTTTAAA
hsa-miR-486-5p	CTGTACTGAGCTGCCCCGAGAA
hsa-miR-148a-3p	TCAGTGCACTACAGAACTTTGAAAA
hsa-let-7b-5p	CGAGTGAGGTAGTAGGTTGTGTGGTTAA
hsa-let-7i-5p	CGAGTGAGGTAGTAGTTTGTGCTGTTAA
hsa-miR-125b-5p	CCCTGAGACCCTAACTTGTGAAA
hsa-miR-122-5p	GTGGAGTGTGACAATGGTGTTTGAAA
hsa-miR-26a-5p	TTCAAGTAATCCAGGATAGGCTAAA
U6	F: CTCGC TTCGGCAGCACA<br>R: AACGCTTCACGAAT TTGCGT

Availability of data and materials

MiRNA-seq data of ADSC-exos generated in this study can be accessed via Genome Sequence Archive, The assigned accession of the submission is: HRA002994 (https://bigd.big.ac.cn/gsa-human/browse/HRA002994). The datasets of current study are available from the corresponding author for reasonable request.
